# Reduction of acetylcholine in the hippocampus of hippocampal cholinergic neurostimulating peptide precursor protein knockout mice

**DOI:** 10.1038/s41598-021-01667-8

**Published:** 2021-11-11

**Authors:** Yuko Kondo-Takuma, Masayuki Mizuno, Yo Tsuda, Yuta Madokoro, Kengo Suzuki, Toyohiro Sato, Hiroshi Takase, Yuto Uchida, Ken-ichi Adachi, Hideki Hida, Cesario V. Borlongan, Noriyuki Matsukawa

**Affiliations:** 1 Department of Neurology, Nagoya City University Graduate School of Medical Sciences, Mizuho-ku, Nagoya, Aichi 467-8601 Japan; 2 Core Laboratory, Nagoya City University Graduate School of Medical Sciences, Mizuho-ku, Nagoya, Aichi 467-8601 Japan; 3 Department of Neurophysiology and Brain Science, Nagoya City University Graduate School of Medical Sciences, Mizuho-ku, Nagoya, Aichi 467-8601 Japan; 4 Center of Excellence for Aging and Brain Repair, Department of Neurosurgery and Brain Repair, University of South Florida, Morsani College of Medicine, 12901 Bruce B. Downs Blvd, Tampa, FL 33612 USA

**Keywords:** Neuroscience, Psychology

## Abstract

The cholinergic efferent network from the medial septal nucleus to the hippocampus plays an important role in learning and memory processes. This cholinergic projection can generate theta oscillations in the hippocampus to encode novel information. Hippocampal cholinergic neurostimulating peptide (HCNP), which induces acetylcholine (Ach) synthesis in the medial septal nuclei of an explant culture system, was purified from the soluble fraction of postnatal rat hippocampus. HCNP is processed from the N-terminal region of a 186-amino acid, 21-kDa HCNP precursor protein, also known as Raf kinase inhibitory protein and phosphatidylethanolamine-binding protein 1. Here, we confirmed direct reduction of Ach release in the hippocampus of freely moving HCNP-pp knockout mice under an arousal state by the microdialysis method. The levels of vesicular acetylcholine transporter were also decreased in the hippocampus of these mice in comparison with those in control mice, suggesting there was decreased incorporation of Ach into the synaptic vesicle. These results potently indicate that HCNP may be a cholinergic regulator in the septo-hippocampal network.

## Introduction

Glutamatergic neuronal activation functions to promote novel information within episodic memory^1,2^. The cholinergic septo-hippocampal neuronal network from the medial septal nucleus (MSN) to the stratum oriens of the CA1–CA3 area plays a crucial role in this process by assisting glutamatergic neuronal activation in the hippocampus, especially under incompetent functioning of the glutamatergic system^3–5^. Cholinergic dysfunction from the MSN to the hippocampus is associated with cognitive abnormalities in a variety of neurodegenerative diseases, such as Alzheimer’s disease or neuropsychiatric diseases^6–8^. Atrophy of the cholinergic basal forebrain is observed even at the prodromal phase in patients with Alzheimer’s disease^9,10^, while cholinesterase inhibitors such as donepezil, rivastigmine, and galantamine hydrobromide have been reported to show clinical effects in terms of cognitive amelioration in patients with Alzheimer’s disease^11–13^. However, the system that regulates acetylcholine (Ach) synthesis in the cholinergic septo-hippocampal neuronal network remains to be elucidated in vivo.

Hippocampal cholinergic neurostimulating peptide (HCNP), which increases Ach synthesis in explant cultures of medial septal nuclei, was identified from the soluble fraction of young adult rat hippocampus in 1992^14^. HCNP is aligned at the N-terminal region of the 21-kDa HCNP precursor protein (HCNP-pp)^15^ composed of 186 amino acids, also known as Raf kinase inhibitory protein (RKIP) and phosphatidylethanolamine-binding protein 1 (PEBP1)^16,17^. In in vitro analyses, kinetic studies demonstrated that HCNP can augment the production of choline acetyltransferase (ChAT) without altering its biochemical affinity^15^. We recently generated HCNP-pp conditional knockout (HCNP-pp KO) mice by using Cre recombinase fused to a mutated ligand-binding domain of the human estrogen receptor, which was driven by a calmodulin kinase II (CaMKII) promoter. Because RKIP has an inhibitory effect on Erk signaling, we generated mice with conditional gene knockouts using the CreERT/loxP recombination system to avoid lethality. We demonstrated that HCNP-pp KO mice show diminished cholinergic projection to CA1. Additionally, the theta activity in CA1 of the hippocampus was reduced in HCNP-pp KO mice^18^. This electrophysiological data suggest that HCNP and/or HCNP-pp is involved in generation of theta oscillations in the hippocampus, which entrains the theta oscillation of the MSN via enhanced ACh release in the medial septum and the enhanced release of ACh in the CA1 stratum oriens^19,20^. However, we were unable to demonstrate reduction of Ach release in the hippocampus of those mice, and alteration of cholinergic neurons in the MSN of those mice. Here, we confirmed the direct reduction of Ach concentration in the ventral hippocampus of freely moving HCNP-pp KO mice under arousal state by using the microdialysis method. Whereas no significant difference in number of ChAT positive neurons in the MSN was shown, the vesicular acetylcholine transporter (VAchT) levels in the hippocampus of those mice were also lower than those in the littermate control mice (Control), suggesting decreased incorporation of Ach into synaptic vesicles.

## Results

### Reduction of Ach concentration in the ventral hippocampus of HCNP-pp KO

**Figure 1:**
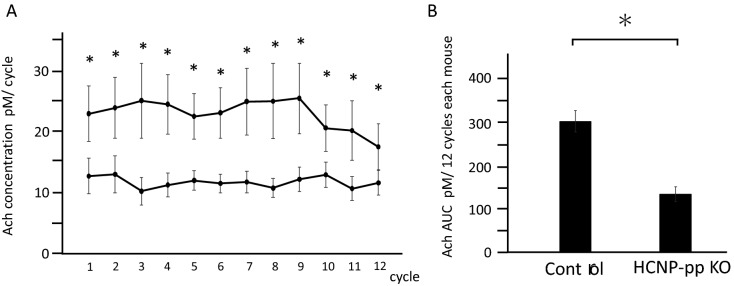
Ach assay using the microdialysis method. (**A**) Time course of Ach release into the extracellular fluid at intervals of 20 min over four hours. At all points, Ach release is lower in the hippocampus of the HCNP-pp KO mice in comparison with that in the Control mice (repeated measure ANOVA: F_(1,191)_ = 4.06, *P* = 0.001; post-hoc analyses with the Scheffe test: 1. F_(1,15)_ = 5.63, *P* = 0.0325, 2. F_(1,15)_ = 5.27, *P* = 0.0376, 3. F_(1,15)_ = 6.56, *P* = 0.0226, 4. F_(1,15)_ = 9.58, *P* = 0.0079, 5. F_(1,15)_ = 12.71, *P* = 0.0031, 6. F_(1,15)_ = 11.95, *P* = 0.0038, 7. F_(1,15)_ = 7.48, *P* = 0.0161, 8. F_(1,15)_ = 6.48, *P* = 0.0234, 9. F_(1,15)_ = 6.59, *P* = 0.0224, 10. F_(1,15)_ = 5.62, *P* = 0.0326, 11. F_(1,15)_ = 4.74, *P* = 0.0410, 12. F_(1,15)_ = 4.63, *P* = 0.0472). (**B**) The Ach area under the curve (AUC) determined by the sum of the concentrations in 12 cycles is significantly lower in the hippocampus of the HCNP-pp KO mice than in the Control mice. Data are presented as the mean ± SEM. Asterisk: *P* < 0.05. Control mice (n = 8), HCNP-pp KO mice (n = 8).

To confirm the involvement of HCNP in the synthesis of Ach in the septo-hippocampal neuronal network in in vivo, we monitored the concentration of Ach in the ventral hippocampus of freely moving mice under arousal state by using the microdialysis method. The dialysate was sampled at an interval of 20 min. The production of Ach in the ventral hippocampus of HCNP-pp KO mice showed a sustained decreased over four hours in comparison with that in control mice (Repeated measure ANOVA, Post-hoc analysis with the Scheffe test; *P* < 0.05) (Fig. 1A,B).

### No significant change was observed in the number of ChAT-positive neurons in the medial septum nuclei of HCNP-pp KO mice

**Figure 2:**
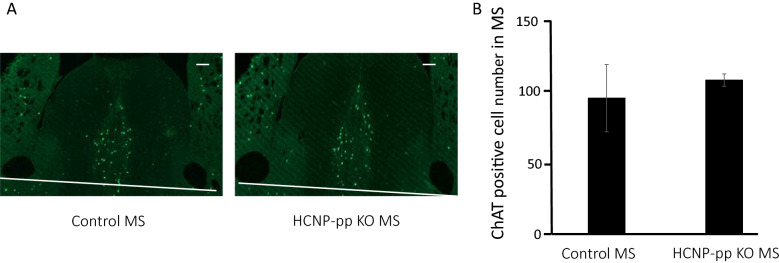
The number of ChAT-positive cells in the MSN is not significantly different between HCNP-pp KO mice and control mice (*P* = 0.6477). The Bayes factor is 122.7, which confirms that the groups do not differ significantly from each other. (**A**) Immunohistochemical analysis using the ChAT antibody. (**B**) Number of ChAT-positive neurons counted on three sequential slice sections of the MSN. Data are presented as the mean ± SEM. Scale bar (right upper) = 100 μm. Control mice (n = 3), HCNP-pp KO mice (n = 3).

We had previously demonstrated a reduction in ChAT-positive axonal terminals in the stratum oriens of the hippocampus of these model mice in comparison to those of control mice by using IMARIS analysis, although western blots could not reveal significant differences between two groups^18^. However, we purified HCNP by using Ach synthesis as an indicator for screening in explant cultured septal nuclei^14^. To confirm the potential of HCNP as a neurotrophic factor for septal cholinergic neurons, we counted the number of ChAT-positive neurons in serial sections of medial septal nuclei. Unexpectedly, there was no significant intergroup difference in the number of ChAT-positive neurons in the medial septal nuclei (Wilcoxon rank-sum test) (Fig. 2A,B).

### Decreased VAchT amount in the hippocampus of HCNP-pp KO mice

**Figure 3:**
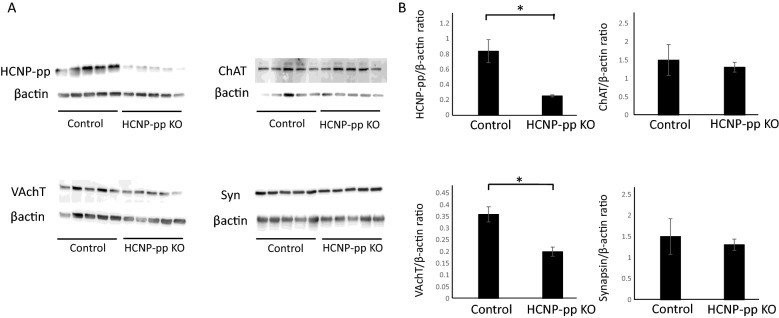
(**A**,**B**) Western blots of ChAT, VAchT, synaptophysin, and HCNP-pp. VAchT concentration is significantly reduced in the hippocampus of HCNP-pp KO mice in comparison with that in control mice, whereas ChAT and synaptophysin concentrations show no significant difference between the groups. Data are presented as the mean ± SEM. Asterisk: *P* < 0.05. Control mice (n = 5), HCNP-pp KO mice (n = 5).

As indicated by our experimental data, ChAT shows limited alteration in the hippocampus of HCNP-pp KO mice^18^, whereas Ach concentration in the extracellular space in HCNP-pp KO mice was apparently lower than that in the Control mice in the current study. Interestingly, VAchT expressing gene exists in the first intron of the ChAT genomic gene^21,22^. Thus, VAchT expression may be controlled with ChAT by a similar regulatory gene expression system. Next step, to confirm the mechanism underlying the reduction in Ach concentration in the hippocampus, we investigated the amounts of synaptophysin and VAchT, including HCNP-pp and ChAT, in the hippocampus by Western blotting analysis. The amount of VAchT in the hippocampus of HCNP-pp KO mice was significantly lower than that in control mice, while no significant difference of synaptophysin was shown between two groups. Similar to the previous report, we also replicated the reduction of HCNP-pp level and observed no significant change in the level of ChAT between the two groups (Fig. 3A,B).

**Figure 4:**
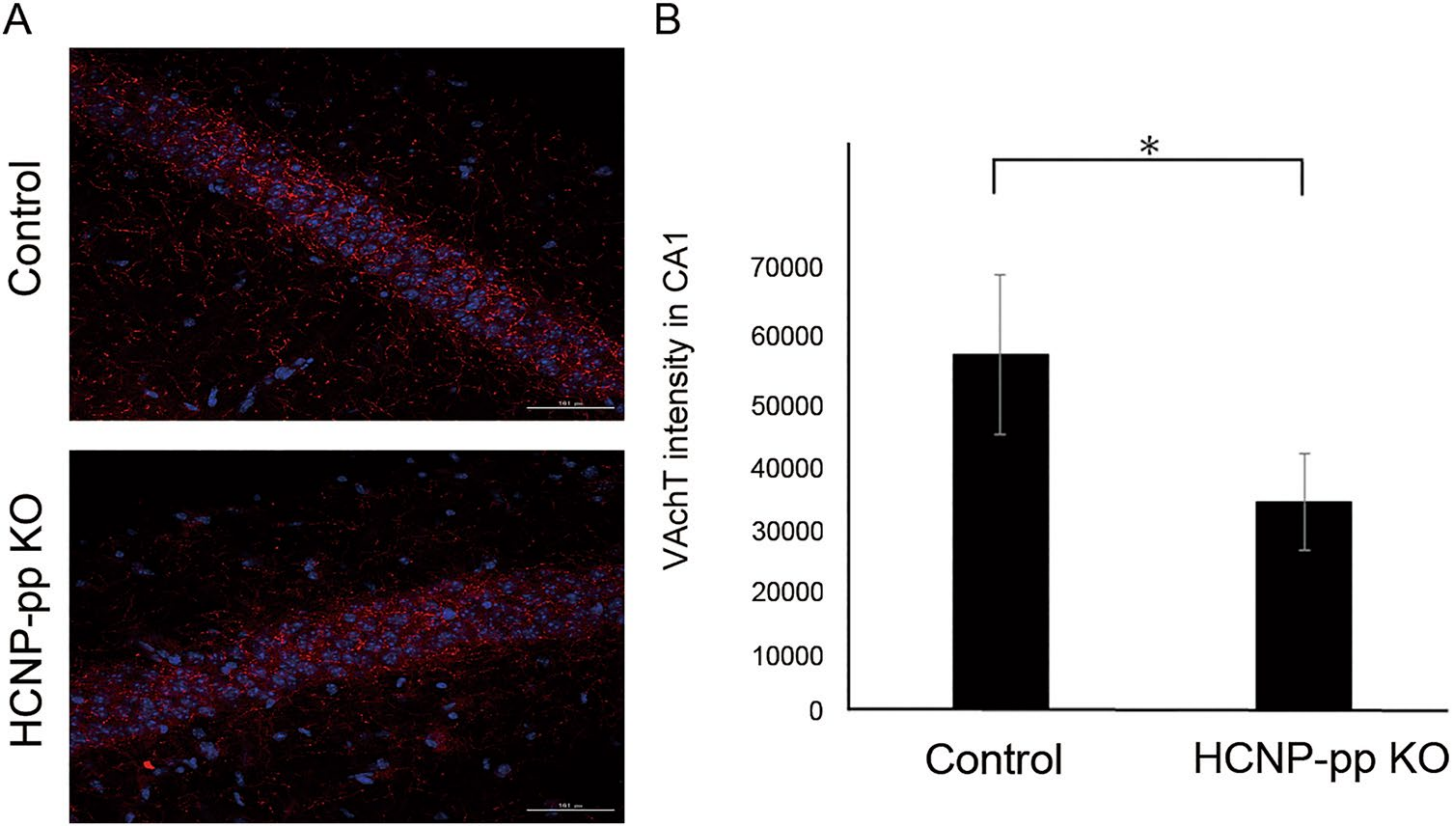
Immunohistochemical findings for VAchT in the hippocampus. VAchT-positive dots are observed densely around the CA1 pyramidal cells, and those in the stratum oriens or stratum radiatum of CA1 are scattered in the hippocampus. Densities of VAchT in the stratum radiatum and stratum oriens are significantly decreased in HCNP-pp KO mice compared to those in Control mice. Data are presented as the mean ± SEM. Asterisk: *P* < 0.05. DAPI; blue, VAchT; red, Scale bar = 100 μm. Control mice (n = 3), HCNP-pp KO mice (n = 3).

Immunohistochemical analysis showed that VAchT-positive synaptic terminals were located densely around the pyramidal neurons and scattered in the stratum oriens and stratum radiatum of the hippocampal CA1–3 area. This suggests that VAchT-positive synaptic terminals in the hippocampal CA1 of HCNP-pp KO mice are significantly decreased in comparison with those of control mice (Student’s t-test; *P* < 0.05) (Fig. 4, supplementary Fig. S1).

### No significant alteration of synaptic number in the stratum oriens and stratum radiatum

**Figure 5:**
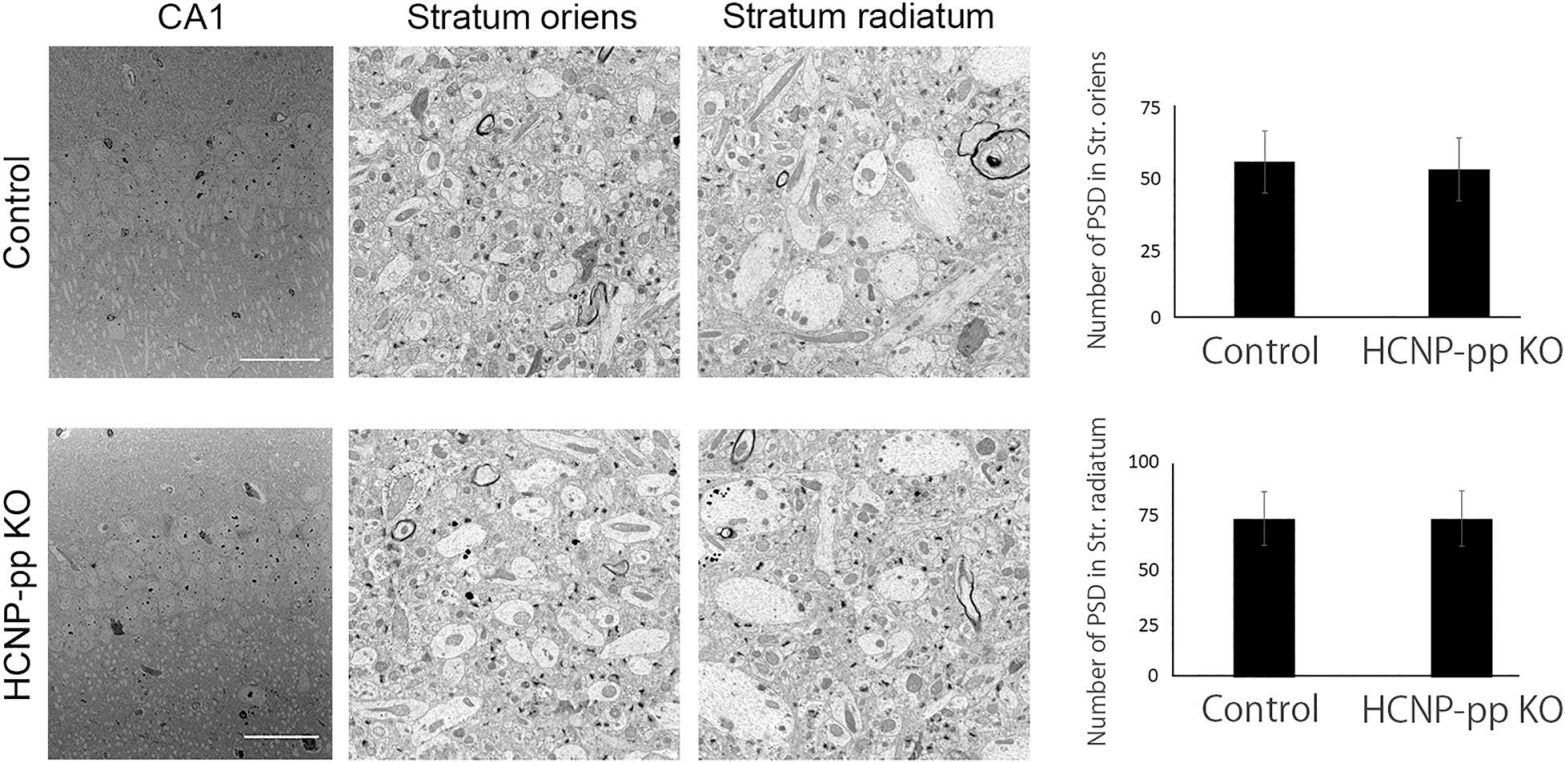
Electron microscopy analysis of the synaptic number in the stratum radiatum and stratum oriens. No significant difference in PSD number is shown in either the stratum oriens or stratum radiatum between HCNP-pp KO mice and Control mice. Data are presented as the mean ± SEM. Control mice (n = 3), HCNP-pp KO mice (n = 3).

To measure the degeneration of synaptic terminals, we performed and analyzed the number of postsynaptic density (PSD) by electron microscope. There was no significant decrease of the PSD number in the stratum oriens and stratum radiatum of hippocampal CA1 in HCNP-pp KO mice as compared with Control mice; cholinergic terminals were specifically unidentifiable (Student’s t-test; *P* < 0.05) (Fig. 5).

## Discussion

Here, we demonstrated that the Ach release into the extracellular space in the ventral hippocampus of HCNP-pp KO mice was significantly lower than that in control mice using the in vivo microdialysis method. The VAchT levels also decreased in the hippocampus of HCNP-pp KO mice in comparison with those in the control mice, suggesting that Ach secretion is inhibited at cholinergic terminals.

HCNP is cleaved from a 21-kDa precursor protein by a specific enzyme of the thiol protease group^15^. In the culture system, HCNP may induce Ach synthesis through a quantitative increase in ChAT levels in the MSN without affecting cholinesterase levels^14^. The amount of ChAT may be also increased in neurons of the MSN in HCNP-pp transgenic mice^23^. HCNP-pp KO mice also presented with diminished ChAT-positive neuronal terminals in the stratum oriens of CA1 and a functional reduction of theta activity in the CA1 of the hippocampus, indicative of inhibition of cholinergic projection from the MSN to the hippocampus^15^. In addition, the amplitude of the hippocampal field excitatory postsynaptic potentials derived by tetanic stimulation, which is mediated through muscarinic (M1) receptor activation, was physiologically enhanced in HCNP-pp transgenic mice relative to that in wild type mice^4^. These electrophysiological data may be consistent with the involvement of endogenous Ach in the enhancement of long-term potentiation (LTP) through M1-mAchRs^24^. These data suggest that HCNP serves as a cholinergic regulator in the septo-hippocampal cholinergic network in in vitro and in vivo. In this current study, we directly confirmed the reduction in Ach release into the extracellular field in the hippocampus of HCNP-pp KO mice compared to that in Control mice, indicating that HCNP functions as a cholinergic regulator for Ach synthesis in the septo-hippocampal network in vivo.

Nerve growth factor (NGF), acting through the TrkA and p75NTR NGF receptors, has been reported to be a potential regulatory factor for Ach synthesis in the septo-hippocampal cholinergic network^25–28^. In cultured explant tissues, HCNP can induce Ach synthesis by increasing the amount of ChAT, while NGF serves as a regulating factor for Ach synthesis by both enhancing ChAT activity and inhibiting Ach esterase activity^15^. In vivo, HCNP may also regulate Ach synthesis in the septo-hippocampal network in conjunction with NGF. The overexpression of NGF or inhibition of the p75NTR NGF receptor protects cholinergic neurons of the MSN in injury models and increases Ach release^29,30^. In the current study, we confirmed that the number of ChAT-positive neurons in the MSN was unchanged in HCNP-pp KO mice compared to that in Control mice, which suggests that HCNP does not performs a trophic function for the survival of cholinergic neurons in the MSN. Thus, HCNP might have a more specific function than NGF in regulating Ach synthesis and/or release in cholinergic neurons.

The septo-hippocampal network acts dynamically through association with multiple transmitters and networks. The input of another transmitter, such as dopamine or GABA, to the septum can also enhance Ach efflux in the hippocampus and participate in learning behavior, which suggests that Ach release is potentially regulated by neural activation in the septo-hippocampal network^31,32^. Cholinergic activation in the MSN can generate theta oscillations, which can entrain theta oscillations in the hippocampus, via direct cholinergic projection and activation of GABAergic neurons in the MSN. We previously demonstrated that the function of GABAergic neurons might be reduced in the hippocampus of HCNP-pp KO mice^33^. In the current study, we showed that the Ach concentration is also reduced in the hippocampus of HCNP-pp KO mice compared with Control mice. Based on our data and previous reports, there is a possibility that the reduction of theta power in HCNP-pp KO mice might be mediated through diminished Ach in the septo-hippocampal formation, which can reduce the activity of GABAergic neurons in the MSN^19,20^.

Ach is synthesized by acetylation of choline using ChAT in the pre-synaptic terminals, and it enters into the synaptic vesicles through VAchT. Membrane potential changes can induce membrane fusion between vesicles and synaptic membranes and release Ach into the synaptic space. After deacetylation of Ach by cholinesterase in the synaptic space, choline reuptake into presynaptic terminals is performed by a high-affinity choline transporter (CHT1)^34^. Treatment with melatonin and *N*-acetyl-5-methoxytryptamine has been shown to inhibit the reduction of the levels of the proteins ChAT, VAchT, CHT1, and M1-mAchRs and improve the cognitive deficit in scopolamine-induced amnesia^35^. NGF also increases VAchT as well as ChAT and CHT1 via the Akt/PKB signaling pathway^36^. HCNP-pp KO mice showed significant VAchT reduction in the hippocampi in comparison with the Control mice, whereas ChAT decrease was limited to the stratum oriens in CA1^18^. The gene locus, including the regulatory element for gene transcription, of VAchT and ChAT is shared, while VAchT and ChAT transcription are coordinately or independently generated^21,22^. Our results suggests that HCNP and/or HCNP-pp may mainly affect the expression of VAchT, in comparison with ChAT, in these HCNP-pp KO mice, while melatonin and NGF equivalently augment VAchT as well as ChAT and CHT1, as described in previous reports^36^.

In the regulation of Ach release, VAchT plays a crucial role in the packaging of acetylcholine into synaptic vesicles. Reduced levels of VAchT in the septo-hippocampal cholinergic network might disrupt hippocampal long-term potentiation^37^. Indeed, the elimination of VAchT in the forebrain of heterozygous VAchT KO mice can provoke dysfunction in spatial memory and social recognition^38,39^. However, HCNP-pp KO, a conditional gene target by Cre-loxP system driven by a CaMKII promoter, is not associated with memory dysfunction and anxiety behavior^18^. To estimate the functions of cholinergic neurons projecting to the hippocampus from the MSN, specific behavior tests for hippocampal function, such as the paired-associate learning task (PAL), are probably needed, which can present spatial memory with disturbances in the synaptic plasticity in the hippocampus of the VAchT limited knockout model^37^.

The limitations of this study include that we could not directly determine whether the amount of Ach in the synaptic vesicle declined, nor could we provide a mechanism to explain the decreased concentration of VAchT in the hippocampus of HCNP-pp KO mice. We also could not confirm whether the amount of ChAT in the cholinergic neurons of the MSN decreased in the hippocampus of HCNP-pp KO mice. Moreover, we did not perform dynamic analyses of CH1 and muscarinic or nicotinic cholinergic receptors, or functional assays of VAchT. Therefore, we cannot exclude the possibility that dysfunction of CH1, together with VAchT, may be involved in the decrease of Ach levels in the hippocampus of HCNP-pp KO mice. In the EM analysis, we could not confirm whether there were changes specifically in the cholinergic terminals in the hippocampi of HCNP-pp KO mice. In addition, the microdialysis analysis evaluated the Ach concentration using artificial cerebrospinal fluid containing an AchE inhibitor to achieve sufficient measurement sensitivity; thus, Ach was not subjected to the natural turnover that occurs in the hippocampus. Further experiments are needed to directly determine the mechanism underlying the reduction of Ach concentration in the hippocampus of HCNP-pp KO mice.

In conclusion, we confirmed that there is a lower Ach concentration in the hippocampus of HCNP-pp KO mice by using microdialysis methods, which suggests that HCNP is a regulator of Ach synthesis in septo-hippocampal formation in vivo.

## Materials and methods

All experiments were performed in accordance with ARRIVE guidelines.

### Animals

Animal experiments were approved by the Animal Care and Use Committees of Nagoya City University Graduate School of Medical Sciences (permit number 18149, 19-017H02) and conformed to the guidelines for the use of laboratory animals published by the Japanese government (Law No. 105, October 1973).

The generation of HCNP-pp KO mice was performed as reported previously^18^. The animals were housed in specific pathogen-free conditions with a 12-h light/dark cycle (lights on 08:00 to 20:00) and given free access to food and water. We used 16 female mice for the microdialysis experiment (age, 50–60 weeks; 8 Control mice and 8 homozygous HCNP-pp KO mice). In 10 of these 16 female mice, the hippocampi contralateral to the side used for microdialysis were used for western blot analyses (5 Control mice and 5 homozygous HCNP-pp KO mice), and the remaining 6 mice brains were used for immunohistochemical analysis of ChAT staining on the MSN, VAchT staining on hippocampi, and electron microscopy on the hippocampus (3 Control mice and 3 HCNP-pp KO mice).

### Antibodies

We generated the rabbit polyclonal anti-mouse/rat HCNP (HCNP-pp) antibody as described previously^15^. The anti-HCNP-pp antibody was purified with an HCNP affinity column prepared with a HiTrap NHS-activated HP Column (GE Healthcare, Waulesha, WI). The following antibodies were obtained commercially: rabbit polyclonal anti-goat ChAT antibody (Merck-Millipore, Billerica, MA, USA)^40^, rabbit monoclonal anti-mouse synaptophysin antibody (Abcam, Cambridge, UK)^41^, rabbit polyclonal anti-mouse VAchT/SLC18A3 antibody (NOVUS Biological, CO, USA)^42^, and mouse monoclonal anti-β-actin antibody (Sigma, MO, USA)^43^ for the western blot, and rabbit polyclonal anti-mouse VAchT antibody (Synaptic System, Gottingen, Germany)^44^ for immunohistochemistry.

### Microdialysis

Mice were anesthetized by intraperitoneal injection of ketamine (74 mg/kg) and xylazine (10 mg/kg) and placed in a stereotactic frame. During surgery, mice were placed on a heating pad. Local anesthesia (2% lidocaine, 2% xylocaine jelly; AstraZeneca, Osaka, Japan) was applied to the skin above the skull before making an incision to expose the skull surface. The skin was disinfected with 70% alcohol and the skull was exposed and cleaned. The guide cannula of the microdialysis probes with an exchange length of 1 mm (A-I-4-1 type; Eicom Corporation, Japan) was implanted in the ventral hippocampus with the following coordinates; from the bregma: AP, − 3.1 mm, L, 2.5 mm; from brain surface: DV, 1.0 mm. Then, the probes were firmly attached with dental cement (Fujilute BC; GC, Tokyo, Japan, Bistite II; Tokuyama Dental, Tokyo, Japan). Mice were allowed to recover for 2 weeks before the dialysate was sampled. Two weeks after the probe implantation, experiments were performed over a day of free movement. The microdialysis probes were perfused for a minute with artificial cerebrospinal fluid (147 mM Na^+^, 4 mM K^+^, 2.3 mM Ca^2+^, 155.6 mM Cl^−^) (Microdialysis manuals, Eicom Japan, Kyoto, Japan) containing 100 nM eserin (FUJIFILM Wako Pure Chemical Corporation, Japan), cholinesterase inhibitor, and 12.5 nM isopropylhomocholine (IPHC) (Eicom Japan, Kyoto, Japan), an internal standard material, at a perfusion rate of 1 µL/min^31,32^. The dialysate was sampled for one minute at intervals of 20 min over 4 h and automatically injected into a microbore HPLC-ECD Eicom HTEC-500 system (Eicom Japan, Kyoto, Japan) in order to measure Ach. During the microdialysis experiment, all mice were awake and able to move freely in the cage. The animals were sacrificed by decapitation after isoflurane anesthesia, and their brains were extracted. After preparation of coronal sections, the correct microdialysis probe location was verified.

### Determination of Ach concentration

The amount of Ach in the dialysates was determined by microbore HPLC-ECD using the Eicom HTEC-500 system, which was equipped with a low-speed pump, pre- and separation columns, an enzyme reactor carrying immobilized AChE and choline oxidase, and an electrochemical detector with a platinum electrode operating at 500 mV. The mobile phase consisted of KHCO_3_ 50 mmol/L (FUJIFILM Wako Pure Chemical Corporation, Kyoto, Japan), EDTS-2Na 134.3 µmol/L (Doujin Laboratories, Kumamoto, Japan), and sodium decane-1-sulfonate 1.64 mmol/L (Tokyo Chemical Industry, Tokyo, Japan) in HPLC gradient grade water brought to pH 8.2. The flow rate was 250 µL/min, with 12.5 nM IPHC serving as the internal control. At an injection volume of 25 µL, the detection limit of this system was 1–2 fmol/injection. Intra-assay and inter-assay coefficients of variability were determined in accordance with a manual for the microdialysis assay^45^. Data acquisition was performed using the EPC-500 PowerChrom R software (Eicom Japan, Kyoto, Japan).

### Western blot analysis

Western blot was performed following previous description^18^. In brief, under deep pentobarbital anesthesia after microdialysis experiments, each mouse was transcardially perfused with PBS (Control: n = 5, HCNP-pp KO: n = 5). After the brains were removed and placed on ice, right hippocampi were dissected and immediately frozen in liquid nitrogen and stored at − 80 °C until use. Frozen hippocampi from each of the five homozygous HCNP-pp KO mice and five control mice were homogenized in four volumes of lysis buffer containing 30 mM Tris–HCl (pH 8.5), 7 M urea, 2 M thiourea, 4% w/v CHAPS, and a protease inhibitor cocktail (Roche Applied Science, Indianapolis, USA). After incubation for 60 min on ice, the homogenates were centrifuged at 15,000×*g* for 3 min at 4 °C. After the protein content was measured with the Bradford assay (Pierce, Rockford, USA), the supernatants were stored at -80 °C until use. Ten micrograms of each supernatant fraction were loaded onto each lane of 10% SDS-PAGE gels. After electrophoresis, the samples were transferred to Hybond-P membranes (GE Healthcare, Tokyo, Japan) using 25 mM Tris, 192 mM glycine, 0.1% SDS, and 10% methanol as transfer buffer. The membranes were then incubated with 1:5000 rabbit polyclonal anti-mouse/rat HCNP (HCNP-pp) antibody, 1:500 rabbit polyclonal anti-mouse ChAT antibody, 1:100,000 rabbit polyclonal anti-synaptophysin antibody, 1:100 rabbit polyclonal anti-VAchT antibody, or a 1:50,000 mouse monoclonal anti-β-actin antibody, and subsequently probed with HRP-conjugated anti-rabbit or anti-mouse IgG antibody. The immunoreactive bands were visualized using the ECL Advance Western Blotting Detection kit (GE Healthcare, Tokyo, Japan) and recorded using ImageQuant LAS 4000 (GE Healthcare, Tokyo, Japan). The western blots were quantified using Amersham Imager 600 Analysis Software (GE Healthcare, Tokyo, Japan).

### Immunohistochemistry

We performed immunohistochemistry following previous report^18^. In brief, after fixation in 4% paraformaldehyde in phosphate buffer (PB, pH 7.4), the brains (Control: n = 3, HCNP-pp KO: n = 3) were equilibrated in 30% sucrose solution in PB and sectioned at 20 μm by using a cryostat (Leica Microsystems, Bensheim, Germany). The sections were incubated overnight with the anti-HCNP-pp antibody (1:500), the anti-ChAT antibody (1:200), or the anti-VAchT antibody (1:4000) in 1% BSA/PBST at 4 °C. Bound antibodies were detected with an Alexa Fluor 488-conjugated or an Alexa Fluor 594-conjugated donkey anti-rabbit IgG secondary antibody (Thermo Fisher Scientific, Japan). Fluorescent signals were detected with an A1Rsi laser confocal microscope (Nikon, Tokyo, Japan).

### Morphological analysis

The density of VAchT immunoreactivity was estimated and analyzed. Briefly, a bregma level of – 2.6 mm in the atlas of Paxinos was selected for semi-quantitative analysis. For the quantification of intensity, three serial sections in each individual from 3 control and 3 HCNP-pp KO mice were imaged by an A1Rsi laser confocal microscope. The images were converted to 256-level greyscale and quantified for the staining intensity per photo using Image J software (Image J, National Institutes of Health, Bethesda, MD).

### Electron microscopy

Electron microscopy was performed as described previously^46^. Briefly, 3 Control and 3 HCNP-pp KO mice were deeply anesthetized and transcardially perfused with PBS followed by a fixative containing 4% paraformaldehyde and 0.1% glutaraldehyde in PB. After trimming hippocampal tissues, the specimens were fixed with 1% osmium tetroxide, and embedded in Epon. The ultrathin sections (90 nm thick) were cut serially with a diamond knife on a Reichert–Jung Ultracut E (Leica, Germany), post-stained with uranyl acetate. Sections were imaged using an electron microscope (JEM-2010; JEOL, Japan). After processing the images, the number of PSDs were counted in each of five regions of interest in the stratum oriens or stratum radiatum from each individual (Control = 3, HCNP-pp KO = 3).

### Data analysis

Statistical analyses were performed using Stata 16.0 (StataCorp, College Station, TX, USA). Continuous variables were calculated as mean ± SEM, based on the normality of data distribution, which was assessed using the Shapiro–Wilk test. The differences in Ach concentration between groups (control mice *vs*. HCNP-pp KO mice) were analyzed by repeated-measures analysis of variance. In assessments of the western blot findings and synapse numbers, data were calculated as the mean ± SEM and analyzed using Student’s t-test or Wilcoxon rank-sum test. To confirm the validity of the comparative statistics, the Bayes factors were also calculated. *P* < 0.05 was considered statistically significant.

## Supplementary Information

Supplementary Figure S1.

## Abbreviations

HCNP: Hippocampal cholinergic neurostimulating peptide
HCNP-pp: Hippocampal cholinergic neurostimulating peptide precursor protein
Ach: Acetylcholine
ChAT: Cholineacetyltransferase
VAchT: Vesicular acetylcholine transporter
RKIP: Raf kinase inhibitory protein
PEBP1: Phosphatidylethanolamine-binding protein 1
MSN: Medial septal nucleus
