# Supplementary material for: Reduction of acetylcholine in the hippocampus of hippocampal cholinergic neurostimulating peptide precursor protein knockout mice
Source: Sci Rep. 2021 Nov 11;11:22072. doi: 10.1038/s41598-021-01667-8 (PMC8586363; doi:10.1038/s41598-021-01667-8)
Supplement: Supplementary file 1 — Supplementary Figure S1. [file 41598_2021_1667_MOESM1_ESM.pptx]

## Slide 1
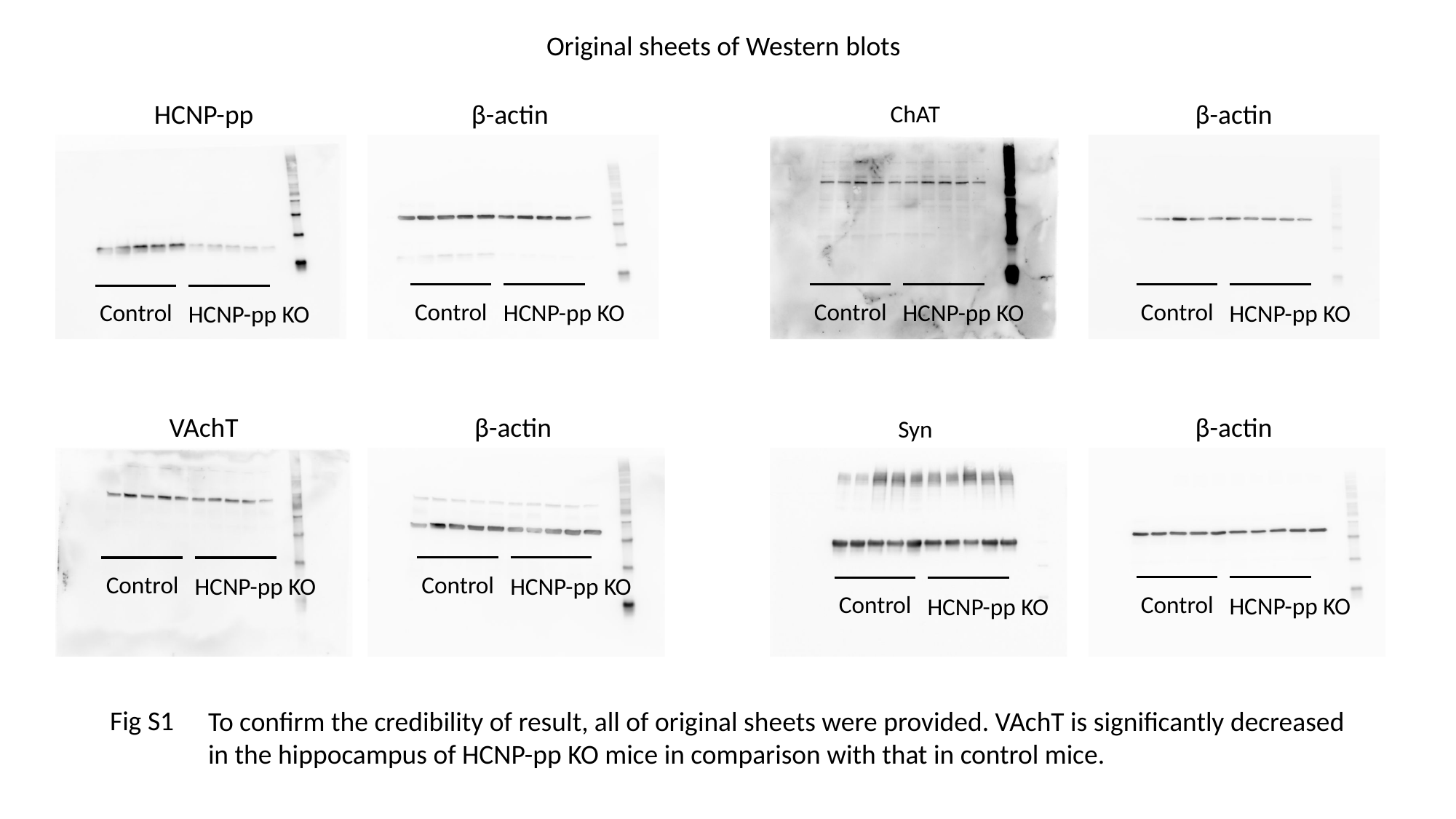

Original sheets of Western blots
HCNP-pp
β-actin
β-actin
ChAT
Control
Control
Control
Control
HCNP-pp KO
HCNP-pp KO
HCNP-pp KO
HCNP-pp KO
VAchT
β-actin
β-actin
Syn
Control
Control
HCNP-pp KO
HCNP-pp KO
Control
Control
HCNP-pp KO
HCNP-pp KO
Fig S1
To confirm the credibility of result, all of original sheets were provided. VAchT is significantly decreased
in the hippocampus of HCNP-pp KO mice in comparison with that in control mice.
